# Entropy, Free Energy, and Symbolization: Free Association at the Intersection of Psychoanalysis and Neuroscience

**DOI:** 10.3389/fpsyg.2020.00366

**Published:** 2020-03-17

**Authors:** Thomas Rabeyron, Claudie Massicotte

**Affiliations:** ^1^Interpsy, Université de Lorraine, Nancy, France; ^2^University of Edinburgh, Edinburgh, United Kingdom; ^3^Young Harris College, Young Harris, GA, United States

**Keywords:** free association, psychoanalysis, symbolization, neuropsychoanalysis, free energy, entropy, primary processes

## Abstract

Both a method of therapy and an exploration of psychic reality, free association is a fundamental element of psychoanalytical practices that refers to the way a patient is asked to describe what comes spontaneously to mind in the therapeutic setting. This paper examines the role of free association from the point of view of psychoanalysis and neuroscience in order to improve our understanding of therapeutic effects induced by psychoanalytic therapies and psychoanalysis. In this regard, we first propose a global overview of the historical origins of the concept of free association in psychoanalysis and examine how Freud established its principles. Then, from Freud’s distinction between primary and secondary processes, we proceed to compare the psychoanalytic model with research originating from cognitive psychology and neuroscience. The notions of entropy and free energy appear particularly relevant at the intersection of these different domains. Finally, we propose the notion of symbolizing transmodality to describe certain specificities of symbolization processes within free association and we summarize the main functions of free association in psychoanalytic practices.

## Introduction: Free Association as the Cornerstone of Psychoanalytic Practices

The effectiveness of psychoanalysis and psychodynamic approaches in the treatment of mental disorders has been the object of numerous empirical studies (Shedler, 2010; Steinert et al., 2017). Current work aims to understand the way such approaches operate, what distinguishes them from other therapeutic methodologies, and their efficacy for long-term psychic transformations (Leuzinger-Bohleber et al., 2019; Woll and Schönbrodt, 2019). Free association – presented by Freud (1913) as the “fundamental technical rule” of psychoanalysis – is often considered as the cornerstone of psychoanalytic practices (Bollas, 2008). Barratt (2016, 2017) thus reminds that “Freud continued to assert consistently that the method of free association is the *sine qua non* of his discipline” (2017, p. 39) and proposes a return to the discipline’s roots relying on free associative praxis. Similarly, for Scarfone (2018), “Free association is really a most distinctive and foundational part of the procedure we call psychoanalysis” (p. 468). Free association thereby appears to be a key concept to examine the modalities and effects of psychic transformation proceeding from psychoanalysis and psychodynamic therapies. In these settings, free association defines the way the patient may spontaneously and unreservedly say anything that comes to mind. The clinician will then be attentive to the way in which the patient goes from one representation to another with more or less fluidity during the therapeutic sessions.

Following these previous lines of research, this article proposes a synthesis concerning the fundamental value of free association processes during psychoanalysis and psychoanalytic psychotherapies. It first presents the historical origins of the concept of free association in psychoanalytic theory, then discusses its development within research in cognitive psychology (Kahneman, 2003, 2011), neuroscience (Friston, 2009; Carhart-Harris and Friston, 2010), and neuropsychoanalysis (Solms and Turnbull, 2011). Despite significant distinctions within these models, we focus on the connections between psychoanalytic and neuroscientific concepts to highlight the heterogeneity of psychic modes of symbolization (Roussillon, 2015), thus developing earlier observations in the fields (Mancia, 2006; De Masi et al., 2015). In this regard, we will underline, as proposed by Cieri and Esposito (2019), how “free association offers a clear and sharp path with cognitive science, free energy neuroscience, and computational psychiatry in order to create a consistent and solid connection between the psychological and neuroscientific views” (p. 5). Free association will thus emerge as a particularly fruitful concept to specify the understanding of therapeutic models through a dialogue between psychoanalysis and neuroscience (Magistretti and Ansermet, 2010; Panksepp and Solms, 2011; Yovell et al., 2015; Rabeyron, 2016).

## Origins of Free Association in Psychoanalytic Therapies and Practices

Historically, reflections on the activity of thought, and the free association which characterizes it, emerged during the 18^th^ century through the “exteriorized” conceptions of Franz-Anton Mesmer. His notion of “animal magnetism” as a “universal flux” that must be harmoniously reordered through various processes (magnetism, passes of hands, etc.) offered a view of mental energy as an external force (Méheust, 1999). This first attempt to represent a “psychic flux” gradually became more “internalized” with the development of psychoanalysis (Laplanche, 1987; Roussillon, 1992). Yet, despite the evolution of psychological theories since Mesmer, the idea of a “flux” that could become “blocked,” thus giving rise to various forms of psychopathology, never completely disappeared, and vestiges of such ideas can still be found in present theories of free association (Roussillon, 2009, 2012; Donnet, 2012).

During the 19^th^ century, Pierre Janet evoked “points of fixation” in psychic activity to describe such obstruction, and Freud (1895) pursued this idea in his *Project for a Scientific Psychology*, yet added the hypothesis that specific “primary defenses” led to these points of fixation. Freud’s originality also consisted in his conception of these defense mechanisms being the consequence of traumas and previous life experiences related to the subject’s affective and sexual life. He explained that an “inhibiting lateral investment” could protect the subject from previous traumatic events by inducing a blockage of free association. This defensive architecture would then limit the patient’s associative capacities^1^. Later, Freud further remarked that these fixations originated from a kernel “of historical truth” (Freud, 1937) – for example, a traumatic experience – which would reemerge through the repetition compulsion because of a “weakness of the power of synthesis” of the ego (Freud, 1941, p. 229).

Freud then supposed that mental functioning and psychopathology could be studied, thanks to free association, according to the particularities of the associative flow and that patients could work through these fixations via free association. He began to use this process with hypnosis and was asking his patients the first words that came to mind while he placed his hand on their forehead. He then conceptualized free association without hypnosis during his work with Emmy Von N. (Freud and Breuer, 1895) and specified his ideas in *The Interpretation of Dreams* (Freud, 1900). Freud showed that the latent content of the dream could be deciphered through the thoughts the patient spontaneously associated with the dream. Freud later used the same technique in *Psychopathology* of Everyday Life (Freud, 1901) to understand slips of the tongue, forgotten words, etc. Then, he employed free association with Freud (1905) to analyze several of her symptoms and again with the Freud (1909) in order to understand the source of the latter’s obsessional behaviors. In his essay *On Beginning the Treatment*, Freud (1913) proposed a clear metaphor to describe the mechanisms of free association to his patients: “Act as though, for instance, you were a traveler sitting next to the window of a railway carriage and describing to someone inside the carriage the changing views which you see outside” (1913, p. 135). For Freud, this method of investigation of psychic reality, and its unconscious processes, also served a therapeutic function and could help the patient release the flow of the activity of thought. During the psychoanalytic treatment, Freud would help the patient to deploy free associations in order to restore or catalyze “blocked” psychological processes and conflicts. Freud’s works about free association thus defined the way in which one passes spontaneously from one idea to another in the psychoanalytical setting and the connections between free association, psychic functioning, psychopathological disorders, and the therapeutic effects of the psychoanalytic treatment.

Thus, it was largely through the free association method that Freud came to analyze the different layers of the psyche and to distinguish between primary and secondary processes corresponding to different “treatments” of psychic energy (Freud, 1915). In the Freudian model, primary processes characterize the unconscious system, while secondary processes are associated with the preconscious-conscious system. In primary processes, psychic energy is said to flow more “freely” and to shape thing-(re)presentations according to the hallucinatory satisfaction of desire. The dream emerges here as a prototype of this type of primary functioning in which operate deformation mechanisms of great malleability, such as displacement and condensation. For Freud, a model of “identity of perception” prevails in primary processes as the psyche appears to reproduce through hallucinations previous pleasant sensorial and perceptive experiences. Within secondary processes, on the other hand, psychic energy has to be bound for the word-(re)presentations to be more stable. The mode of satisfaction appears to become secondarized and “identity of thought” now prevails, for the source of pleasure in secondary processes is no longer the identical reproduction of a previous pleasurable experience but the symbolic thinking associated with the initial pleasant experience. In its relation to the world, the psyche has thus sacrificed part of its freedom in its relation to pleasure in order to adapt to reality, and the associative flux thereby finds itself diminished.

Freud supposed that the analyst should be in a specific state of mind called “free floating attention” while the patient is free associating. In this way, analysts might use their own unconscious to decipher the unconscious of the patient. Contemporary psychoanalytical models of free association have since insisted on this aspect and claim that free association is only fully effective when coupled with this form of free association coming from the analyst. This “shared” free association, or co-associativity^2^ (Roussillon, 2011) implies that the patient associates freely in the presence of the clinician and addresses oneself through the other. Alterity thus emerges as a fundamental dimension of the associative process: one may not freely express the secrets of one’s most intimate psychic life in a solipsistic way; rather, one must find the conditions to deploy free association in the intersubjective relationship (Barratt, 2017).

Various psychoanalysts since Freud have argued that this shared free association operates at a very “primary” level through a form of “co-thinking” (Widlöcher, 1996, 2010) or “co-psycheity” (Georgieff, 2010) particular to psychodynamic psychotherapies and the psychoanalytic setting. Thanks to the transference process, the spontaneous free associations of the analyst may reflect some unelaborated aspects of unconscious processes in the patient’s own associativity. We are thus dealing with an “analytical third,” that is to say a melting of the free association processes of the patient and analyst at a very primary level (Ogden, 1994). Eschel (2006) describes more precisely a process of “twogetherness” constituting a form of “associativity of presence” when the relation to the psychoanalyst is established primarily through affects. This shared and primary associativity becomes the breeding ground necessary for the emergence of a “moment of meeting” (Stern, 2004) during which both clinician and patient feel that a step has been made toward maturation and symbolization processes.

Some yet unmetabolized experiences will then “blister” (*boursoufler*) the patient’s free association and behaviors in the psychoanalytic setting in order to be shared and recognized (Roussillon, 2012; Lothane, 2018). The patient may act out – the Freudian *agieren* – what remains unelaborated from previous sufferings and pathological relationships. For example, this process may give rise to the “fear of breakdown” described by Winnicott (1963), a fear which re-emerges in consequence of early primitive sufferings. It may also occasion the return of traumatic experiences in hallucinatory forms during the therapeutic sessions (Botella and Botella, 1990). These past traumatic experiences will leave “knots” or “marks” on free association, the latter being “directed at unraveling the knots in the patient’s psyche” (Scarfone, 2018, p. 474). The work of integration and transformation operating through the “unbridled” free association in the clinical setting therefore requires that the unelaborated experience be expressed, notably through the transfer, “fragment by fragment,” or “piece by piece” as suggested by Freud (1913). A “transfer” and a shared associativity then allow a translation process of the past traumatic experiences. This process permits the patient to “re-feel” or “re-know” an experience that has remained unmetabolized in order to improve reflexive awareness, which is catalyzed and condensed by the clinical setting. Free association and reflexivity thus share a need to deploy themselves through exteriority: what cannot be represented and symbolized through intrapsychic processes must be “externalized” thanks to free association and the intersubjective relationship in order to be elaborated.

## Free Association and Free Energy From the Point of View of Cognitive Psychology and Neuroscience

To what extent are these models of free association developed in psychoanalysis in line with recent work in the field of cognitive psychology and neuroscience? A first comparison emerges through the work of Kahneman (2003) who focuses on the understanding and modeling of reasoning biases, studying them through various ingenious experiments. Kahneman (2003, 2011) proposes a division of consciousness according to two principal modes of thinking. He calls the first “System 1” to describe reasoning fallacies emerging from a fast and imprecise activity of thought linked to intuitive functioning^3^. This System encompasses automatic feelings and inclinations, is almost instinctive, and yet is shaped by experience. System 1 builds logical causalities outside the sphere of conscious awareness and is easily influenced by phenomena of suggestion and priming^4^. Mood and cognitive engagement also have a major impact on this System’s functioning. System 1 is sensitive to the “halo effects” and produces a set of approximations in reasoning. Kahneman concludes that the human brain naturally favors the slightest effort and prefers to stick to the most accessible information. When the approximations thus produced are not secondarized – that is, validated by System 2 –, subjects tend to make more cognitive mistakes. Kahneman describes System 1 as an “associative machine” functioning through logics of “associative coherence,” in the sense that it spontaneously and automatically constructs meaning from underlying causal links.

Through an original methodology, Kahneman’s research echoes Freud’s attempt at mapping psychic heterogeneity through the distinction between primary and secondary processes. Kahneman and Freud’s approaches may be compared through the following table inspired from Roussillon (2001) and Kahneman (2003).

**Table:** 

System 1 – Primary processes	System 2 – Secondary processes
Quick temporality	Slow temporality
Automatic	Reflexive
Unconscious	Conscious
No negation	Negation
Intuitive	Rational
Perceptive	Conceptual
Pleasure principle	Reality principle
Free energy	Bound energy
High entropy	Low entropy

Although these two models do not overlap entirely, it is interesting that, despite very different methodologies, both Freud and Kahneman find two main “layers” of psychological functioning whose characteristics can be translated from one model to another. We could consider the S1 and S2 described by Kahneman as the expression of primary and secondary processes at a cognitive level of functioning even if distinctions remain: Kahneman analyzes psychic modes of functioning primarily in terms of cognitive and reasoning mechanisms, while Freud presents a theory of the psyche that deals primarily with its psycho-affective construction. One might also add that Freud is asking the question of “why,” while Kahneman focuses on “how” the psyche functions through these two processes. Yet, Freud and Kahneman’s theories converge through their understanding of the fundamental bi-polarity of psychic processes which are often working in concert and which leave their “mark” on mental functioning and free association.

We will now turn to the work of Friston (2009) on the free energy principle (FEP) to describe in more detail a second parallel between Freud’s work on free association and recent research in cognitive neurosciences, knowing that “in the last 10 years, the FEP has become the royal road in the dialogue between neuroscience and psychoanalysis, *the bridge* between mind and brain” (Cieri and Esposito, 2019, p. 3). Freud initially studied the heterogeneity of psychic functioning according to the way in which the psyche needs to bind and connect nervous energy after sensorial stimulation from the environment. In their *Studies in Hysteria (1895)*, Freud and Breuer built upon the theories of contemporary physicists – especially Hermann von Helmholtz – to formulate the distinction between “static” and “kinetic energy,” and Freud developed this opposition through the notions of “free energy” and “bound energy” differentiating the primary and secondary modes of psychic functioning. Later, Freud (1920) supposed that “the primary function of the psychic apparatus was to bind the amount of excitation reaching it” and he conceived neurosis as the consequence of a “surprise” taking the form of a fright induced by traumatic events.

These hypotheses join the recent theories of Karl Friston (2013) who reminds that every living organism must resist the second law of thermodynamics, the spontaneous tendency of any physical system to move toward a state of disorganization, that can be measured through degrees of entropy. Friston (2013) supposes that biological organisms must protect themselves against high degrees of entropy which could result in their death. A high entropy level signals a greater level of disorganization and can come from an external source (for instance, from the environment) or from the organism itself (notably through the natural and spontaneous tendency toward disorganization coming from physical and biological properties of matter)^5^. Also drawing upon Helmholtz’s hypotheses, Friston suggests that the brain obeys the same principles and constantly produces coherent and predictive representations of the external world in order to limit entropy and its own disorganized states. To limit increases of internal disorganization, the brain develops a Bayesian^6^ probabilistic model to determine potential causes of sensations according to prior beliefs and experiences. But this work of prediction is not perfect and sometimes results in a discrepancy between perceptual data from the environment and mental representations supported by the neural network. Friston (2009) calls this discrepancy or disorganization “free energy.” It will induce a subjective feeling of surprise, and since the psyche cannot simulate all possible encounters with the environment, states of “surprise” sometimes arise as the consequence of free energy.

The brain thus constantly responds to interactions with the environment in an enactive way (Ramstead et al., 2019)^7^ and these interactions lead to the development of a “generative model” that allows one to make predictions about the environment. The more reliable these predictions are – or the more the brain limits the gap between the internal world and the external world –the lower is the entropy generated in the brain^8^ and the fewer are the effects of surprise. Friston also posits that this generative model is organized with a hierarchical structure where higher levels of cerebral functioning exert constraints on the lower levels. Thus, “suppressing free-energy means that each level tries to explain away prediction errors at its own level and in the level below” (2009, p. 295). Friston also describes the complex relationships between these hierarchical levels, and the top–down and bottom–up processes that modify neuromodulation and the mechanisms of “associative plasticity” at a biological and synaptic level (2009, p. 300). His theory, framed by the computational model, therefore offers an understanding of the neuronal constraints of associativity depending on the FEP.

**FIGURE 1:**
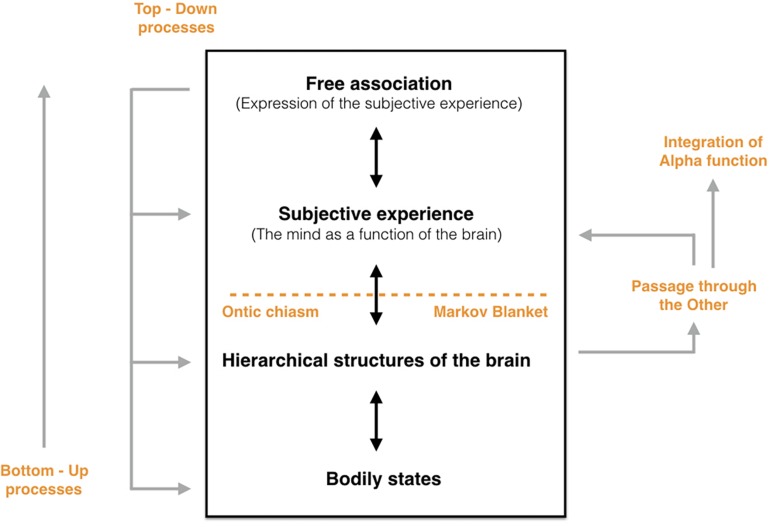
This diagram proposes a synthesis of the passage from the (1) bodily states to (2) the hierarchical structures of the brain to (3) the subjective experience and to (4) the free association that expresses this experience. There exists between the purely biological level and the subjective experience an ontic chiasm which specifies the differences between the processes of conscious psychic functioning. A Markov blanket operates as a space of delimitation and communication between these two levels in the sense that this blanket forms an organizational boundary which allows for the emergence of the subjective experience. This mental functioning emerges through symbolization processes shaped by early intersubjectivity and the passage through the other that characterize it. This intersubjectivity at the origin of thought is gradually internalized and takes the form of what Bion (1965) calls the Alpha function. The highest levels of psychic functioning also influence the lower levels thanks to the top–down processes while the bottom–up processes emerge from the biological levels and give birth to the top–down processes.

Pursuing Friston’s work on free energy and systems theory, Connolly and van Deventer (2017) explain that the FEP operates at various levels of the organism, what these authors refer to as the “scale free principle.” They also draw on Hobson et al. (2014) claim that there is a “hierarchical nature of generative or virtual reality models” (p. 11) to suggest that “the predictive model is organized at multiple nested layers, all of which are influenced by the FEP through this recursive feedback process” (p. 11). But “while psychoanalytic mental processes are fundamentally subject to the FEP, they nonetheless also add their own principles of process over and above that of the FEP” (Connolly and van Deventer, 2017, p. 2). The same authors continue: “the level above (which is psychological) cannot violate the FEP. However […] new organizational principles emerge at this level, so that it is not fully explained by the FEP” (p. 7). Consequently, one cannot understand the highest hierarchical levels solely through the FEP because of the emerging properties of the highest hierarchical level of brain functioning. The organization of these higher-order levels will then affect the lower levels from which they originate and influence the activities at lower levels. Thus, subjectivity and free association appear as a functional flow emanating from the neurological system but developing emergent properties impacting in return the underlying biological systems (see Figure 1). There are therefore multiple levels in the generative model (sensory systems, memory, self-representation, etc.) and each obeys various operating logics according to an increasing degree of complexification^9^. These levels, which affect each other through recursive loops,^10^ communicate and are distinguished by “the existence of a Markov blanket^11^ within the brain [that] affords the opportunity for higher levels in the brain to make inferences about lower levels” (p. 11). How, then, might we study these different levels and which are the most fundamental?

Connolly and van Deventer (2017) offer an interesting response: “it is neither possible nor even desirable to build a complete picture of every possible level of organic and neural organization superordinate to the basic level of biological organization which is the FEP, up to the level of interest which is here psychoanalysis. Rather, it is desirable to identify some of the most significant forms of organization that are foundational to psychoanalysis, but superordinate to the FEP, which can build an intelligible bridge between the two” (p. 12). The authors continue: “What would be needed would be a description of the most relevant and proximal layers that most closely influence the level of interest which is that of psychoanalytic regulatory principles” (p. 13). This is exactly what Freud tried to do by showing the main principles organizing psychic reality (pleasure principle, reality principle, principle of constancy, etc.). Likewise, the distinction between primary and secondary processes appears to make up the two most significant levels of mental functioning associated with specific principles, as suggested by both Freud (1900) and Kahneman (2011). As we shall now explore, Solms, Friston, and Carhart-Harris also propose a model that reflects and enriches psychoanalytic models and the modelization of these principles, particularly as they relate to free association.

## Consciousness, Free Association, and the Default Mode Network

Mark Solms has opened an important dialogue between research in contemporary neuroscience – especially the work of Karl Friston – and psychoanalytic models concerning the notion of free energy. In an article entitled “The Conscious Id,” Solms (2013), following the work of Panksepp (1998, 2010)^12^, criticized the cortico-centric view of the psyche which considers the cortex as the center of consciousness. Solms rather suggests that there is a primary and affective^13^ form of consciousness closely connected with the reticular system^14^ which exists prior to the cerebral cortex^15^. Thus, Solms argues that consciousness depends initially on logics relating to the Freudian id rather than the ego^16^. As for the cortex, its essential function is not to produce consciousness, but to “stabilize” objects of perception, and it is “merely a repository of memory images” (Solms, 2018, p. 6). Mental representations may thus attain preconscious and conscious processes when they are transformed by the cortex into a material sufficiently stable to become the object of working memory. To put it differently, for Solms, “The essential function of the cortex” is to generate “stable, representational ‘mental solids’ that, when activated (or ‘cathected’) by affective consciousness, enable the id to picture itself in the world and to think” (2013, p. 14). The cortex would thus contribute to the emergence of a “space of representational memory” from which free association could be deployed.

Solms also supposes that “free energy minimization is the basic function of homeostasis” and that “the functions of homeostasis and consciousness are realized physiologically in the very same part of the brain” (Solms, 2018, p. 10). Consciousness would then be “an extended form of homeostasis” conducting to a specific functional organization which would represent an adaptive advantage. In Solms (2013)’ model, primary processes appear to belong to a first form of consciousness, characterized mainly by affects, and preceding a secondary form of consciousness whose function is to stabilize mental objects. In other words, the transition from primary processes to secondary processes would correspond to the way in which free energy becomes bound by secondary processes, thus permitting the stabilization of mental representations and their access to a secondary or reflexive form of consciousness (Solms, 2013). But how might this transition from the primary affective consciousness to the secondary consciousness, from free energy to bound energy, arise? And what is the influence of this transition on our understandings of free association?

According to Carhart-Harris and Friston (2010), this transition emerges thanks to the “default mode network” (DMN). They suppose that the DMN is consistent with Freudian ideas of the ego that could take part into this transition from primary to secondary processes. The DMN defines a network that develops during childhood and connects several anatomical zones remaining active during the resting state – notably the medial temporal lobe, the medial prefrontal cortex, the posterior cingulate cortex, the precuneus, and other neighboring regions of the parietal cortex (Buckner et al., 2008)^17^. It consumes more energy than any other areas of the brain, a fact that signals a high associative density between these other areas. For Carhart-Harris and Friston, the activation of the DMN also corresponds to a decrease in the activity of the lower levels of organization, which suggests that it serves to modulate internal and external inputs or to suppress prediction errors (the free energy stemming from lower levels of mental functioning). The DMN is mainly engaged in higher mental operations, such as meta-cognition and reflexivity, as shown by several imaging protocols (Carhart-Harris and Friston, 2010). Spontaneous oscillations in the posterior cingulate cortex, particularly in the alpha of 8–13 Hz, are a neurological marker of the DMN’s functioning that Carhart-Harris and Friston further link to a possible work of integration by the ego (2010, p. 2). Lastly, the activation of the DMN is inversely proportional with the attention system^18^ and its activity appears to decrease with age as well as in people with attention deficit disorders.

From these different elements, Carhart-Harris and Friston hypothesize that the functioning of the DMN offers a neurobiological equivalent to Freud’s ego. More precisely, according to Friston (2009), conscious activity, linked to the processes of the DMN, would constitute a temporary measure of adaptation between the brain and the environment. The brain must attempt to “correct” any discrepancy between the internal model of reality and the external reality. Thus, the fundamental aim of the DMN would be to limit its activity by seeking an “automatic” mode that would minimize the necessary adjustments between internal reality and external reality. Cieri and Esposito (2019) also suppose that “the DMN seems to play the same function of mediation attributed by Freud to the ego and some authors have spoken about *Default Self* in order to define the DMN as a kind of biomarker of the Self” (p. 6). They add that “the DMN is consistent with ego functions and with its target of containing free energy levels of underlying structures, a function of the secondary process. The result is a top–down hierarchy of DMN which aims to reduce the free energy associated with the Freudian primary process” (p. 12). Dimkov (2019) suggests an alternative view in which DMN is co-activated with Centre-Executive Network during regression processes. From his point of view, “DMN appears to function as a third thought process, an intermediary process between the primary and the secondary ones” (p. 170).

**FIGURE 2:**
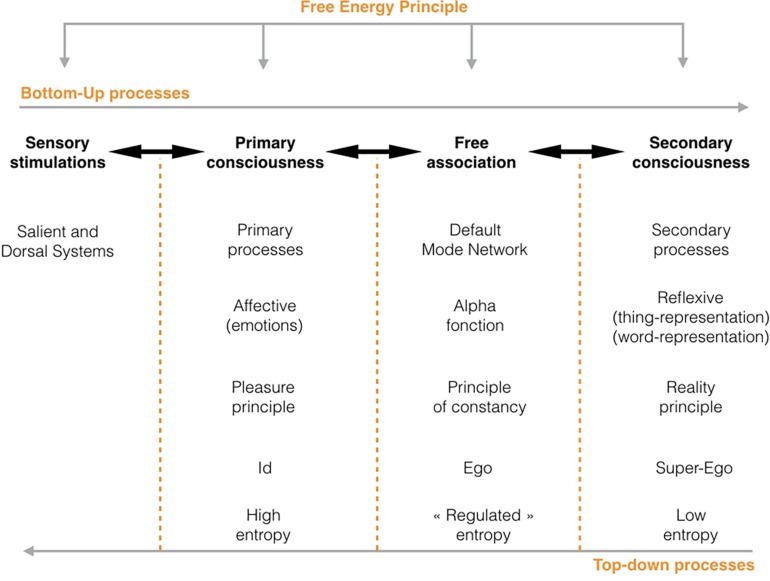
The FEP applies at the different levels of mental functioning from sensory stimulation to secondary consciousness. We have here indicated the major neurobiological processes, the type of processes and principles, the equivalents in the Freudian topography, as well as the level of entropy at each level of functioning. Following Dimkov’s hypothesis, we suggest that the DMN is a process of articulation between primary and secondary processes rather than the expression of the functioning of secondary consciousness. Free association thereby appears as the expression of the work of psychic integration carried out by the DMN. The following diagram does not pretend to represent a “scientific” model of psychic functioning but rather offers a general representation of the logics of functioning of the psyche and the intersections of neuroscience and psychoanalysis.

Solms (2013) argues that this work of articulation and prediction operates during the transition from primary to secondary processes, or from affective to stabilized consciousness^19^. The world becomes more organized and “predictable” as the effects of surprise diminish. The main function of consciousness is then to carry out this work of prediction through the affects self-informing the subject regarding the relevance of its generative model. The primary relation to the world is thus an affective relation intrinsically linked to the pleasure principle. Secondary consciousness serves to “re-work” unrepresented affects arising from a painful discrepancy between internal and external worlds. When the effects of surprise disappear, this form of consciousness is no longer necessary, in the same way that the dancer does not need to reflect upon the movements practiced a thousand times. Linguistic systems (word-representations) allow the subject to regulate these primary affects, opening the way to forms of associativity obeying different principles. Signifiers will thus participate in secondary processes by adding an additional “delay” and reshaping associative processes depending on structural laws of language (Lacan, 1966). Thus occurs a transfer from the primary associative logics to the linguistic apparatus, from affects to word-representations (see Figure 2). The latter, as Roussillon et al. (2007) suggest, will nevertheless keep the “trace” of this passage through the body in the form of a particular “corporeity” or “materiality” of language, showing that one form of associativity does not make the previous one disappear^20^. Free association – expressed linguistically – thus keeps the influence of the subject’s overall psychic functioning, which explains its essential function in the exploration of the patient’s psyche. For the analyst, it is the equivalent of the biologist’s microscope in that it allows for an “inside view” of psychic functioning as it occurs.

The brain thus appears to have evolved in order to simulate its environment and diminish the effects of surprise thanks to a Bayesian model. Free association can be considered as an echo of this process insofar as it reflects the functioning of psychic reality, itself constructed through a constant relation with the environment virtually simulated via a generative model (Hopkins, 2016). The intrapsychic associativity thus preserves the trace of the environment –an “externalized” associativity – to which the subject has been confronted.

## Free Association and the Entropic Brain

Carhart-Harris extends this understanding of associativity with the “entropic brain” theory, which refers to the degree of organization or uncertainty of conscious states (Carhart-Harris et al., 2014). For Carhart-Harris, the hierarchical structures of the brain are situated within a continuum depending on different levels of organization. The primary consciousness described by Solms corresponds to a higher degree of entropy, for it is less “meticulous” in its relation to the world and is highly malleable. The secondary consciousness, on the other hand, works to diminish high entropy levels resulting from the primary consciousness by organizing and constraining cognition. Thus, primary consciousness is more “entropic” and flexible than secondary consciousness, which presents a higher degree of organization and a lower degree of entropy^21^. When the relation to the environment becomes a source of uncertainty or puzzlement, the subject has to “contain” this uncertainty^22^. The subject can then react in different ways: for example, “magical thinking” will interpret the world according to one’s desires (the pleasure principle) when a high level of entropy “overflows” the secondary processes. Depressive states, on the other hand, will demonstrate a difficulty in balancing the uncertainty arising from primary levels of psychic functioning^23^. In such states, neuroimaging has revealed a hyper-activation of the DMN, a consequence of hypertrophied introspection and a desperate attempt of the ego to control the entropy stemming from primary processes. Carhart-Harris describes this movement through the theory of “self-organized criticality,” which shows how a complex system develops specific properties when disturbed to a critical extent by a new energy input (Carhart-Harris et al., 2014). In the narrow transition zone between the extreme positions of chaos and order, three properties will emerge: metastable or transiently-stable states, a sensitivity to perturbation, and a propensity for cascade-like processes called “avalanches.” These could find their correlate in the functioning of the ego and psychopathological expression. For example, avalanche processes could lead to psychotic collapse showing how the ego is suddenly unable to internalize new energy input^24^.

Carhart-Harris et al. (2014) have experimentally studied these variations of organization and associativity through psychedelics – particularly psilocybin – and revealed that the latter produces a prototypical primary state of consciousness with high entropy. Psilocybin alters consciousness through a disorganization of cerebral activity, which translates into a significant decrease in the activity of key brain areas connected to the DMN. Psychedelics can thus generate profound states of insight concerning the self, often referred to as an oceanic feeling (Freud, 1930) of dissolution of the ego and its borders. The phases of paradoxical sleep, initial and acute psychotic periods, and certain epileptic states also seem to engender regression to primary consciousness. Thus, as Freud suggested, dreams and psychoses probably pertain to primary forms of consciousness (also dominating in infancy), while meta-cognition would develop only secondarily (on this topic, see also, Hopkins, 2016).

In sum, a distinction emerges between two main states of cognition, the first being characteristic of the state of consciousness of the adult, and the second, present in infancy, reappearing through mechanisms of regression. These two states of consciousness are related to certain frequencies of neuronal activity, in particular the power of alpha waves correlated with reflexive activity (Carhart-Harris et al., 2014). Certain cerebral rhythms correspond to a decrease in entropy due to an increase in the exchange of information between neural networks. The use of psilocybin, in particular, induces a decrease in the activity of the alpha spectrum, thus resulting in a subjective feeling of disintegration. Under the effects of such psychedelics, the brain behaves more randomly, its hierarchical functioning becomes anarchic, and the associativity becomes more flexible, regressing to primary modes of functioning^25^. The therapeutic effects of psychedelics could therefore rest on an “extreme” form of symbolization^26^ different from the usual, more “attenuated” symbolization found in psychodynamic psychotherapy and psychoanalysis. The deployment of free association and the passage through high entropic states would allow for a necessary relaxation of the psyche, thereby reviving processes of symbolization. Examining free association therefore seems crucial for understanding psychic integration between internal and external worlds.

## Free Association, Symbolization, and Psychoanalytical Practices

To what extent do these theoretical models of free association resonate with, and affect, clinical practice? While the works discussed above essentially focus on normal cognitive functioning, clinicians generally work with the failures of the “associative machine” or try to catalyze symbolization^27^ processes thanks to free association^28^. When the clinician asks the patient to verbalize “everything that comes to mind,” he or she intends to help the latter “untie” the free association that leads to a subjective and complex mix of sensations, emotions, images, words and memories. The patient will then use spontaneously the different “languages” at his or her disposal, such as breathing, motion of the body, facial expressions, words and narratives to share his or her psychic life in the clinical setting^29^. The work of free association therefore follows the different modes of symbolization in order to share, integrate and transform the internal experience within the present therapeutic intersubjective relationship.

A primary form of free association concerns mainly the emotions emerging within the dialogue of therapy. This form of shared consciousness, or this “affective co-consciousness,” relies on primary processes and arises from the clinician’s regressive skills at the most primary levels. As Parat (2013) explains, “This forms the possibility of an inter-human relationship, which is established directly and regressively in a preverbal, ante-verbal mode, and where the affect of one echoes the affect of the other. [This relationship] is perhaps the only way to allow for the approach and mobilization of the elements, the sediments of the primary repression” (p. 171). For Parat, the clinician’s position opens the way to a “basic transfer,” an expression that approaches Stern (2004) “intersubjective sharing” or Christian David (1992) “accompanying activity.” This sharing of affects presents a first form of intersubjective and undifferentiated associativity that permits the release of a non-symbolized psychic “residue.” As de M’Uzan (1994) work on “paradoxical thought” (*chimère*) and Widlöcher (1996) concept of “systems of co-thinking” demonstrate, this primary associativity can be particularly “permeable,” as it is characterized by psychic transmissions from unconscious to unconscious (Evrard and Rabeyron, 2012). The psychotherapeutic dyad thus produces an “analytic third” (Ogden, 1994) which combines the indissociable thoughts of patient and clinician^30^.

At a more elaborate level of psychic functioning, the passage through words, to form new signifying chains, breaks this primary and shared form of regression. In other words, there emerges a “secondary associativity” that requires the patient to come out of his or her state of regression and to integrate the experience at higher levels of functioning. This work operates more specifically through conscious activity and word-representation, and it reduces the entropy coming from lower levels of mental functioning. The patient may then deploy more elaborated and secondary levels involving the stabilization of mental objects, as Solms (2013) suggests. This work of stabilization may participate in the process of psychic integration as evidenced, for example, by the patient who suddenly becomes able to understand previously unintelligible parts of his or her experience (affects, behavior, etc.). It produces what Freud (1937) called a “construction” – or what Bion (1965) named a “selected fact” – that re-organizes experiences through the medium of speech. Here, perhaps in ways similar to the processes of “reconsolidation” of memory traces described by Alberini et al. (2013), the raw experience can then be treated again through the different levels of associative processes as the linguistic apparatus comes to regulate primary processes. Words then come to the rescue of the body and the unrepresented affect.

Free association thus emerges as an essential component of this work of symbolization at the intersection of primary and secondary processes. It permits the subject to diminish its investment in the external environment in order to increase attention to intrapsychic reality. Akin to the caterpillar metamorphosing in its cocoon, the patient can here safely elaborate the experiences that have not been integrated in the psyche (Rabeyron, 2019). Free association thereby augments the free energy that was, until then, contained by defense mechanisms such as repression and splitting. Free association increases the prevalence of primary processes, thereby occasioning regressive and hallucinatory states^31^. This regression to primary processes truly allows for psychic integration to occur when coupled with secondary processes as a complementary system necessary for the reflexive metabolization of the subjective experience. This requires a very particular psychic activity which underlies the effects of free association and which corresponds to what Bion (1965) calls the “alpha function” permitting the transformation of sensations and emotions into thinkable contents. Freud (1900) had already intuited this function in suggesting that the dream was necessary for the passage from primary to secondary processes. Bion (1965), however, demonstrated that the dream work is always present in the psyche. We “dream” both day and night insofar as we constantly need to transform our experiences into subjective psychic matter. The distinction between thinkable and unthinkable thoughts emerges in the passage through the alpha function which distinguishes, by a membrane, the conscious and unconscious processes.

The DMN could be a neurobiological equivalent of the alpha function described by Bion^32^, a function that bridges various levels of psychic integration and more particularly the primary and secondary processes^33^. The work of psychic integration requires both the dream regression and the elaboration of past experiences. Bion (1965) model also insists on the idea that the alpha function results from the integration of the alpha function of the mother. This function involves three factors: daydreaming, diffraction-synthesis and contained-container. The first of these factors – daydreaming – occurs as the mother takes care of the child and is the prototype of the alpha function. The work of articulation between psyche and soma, between the unconscious and consciousness, therefore emerges from the early intersubjective relation between the baby and the mother. This long and complex process may explain why subjectivity, from a psychoanalytical point of view, takes many years to emerge in the human being.

The emergence of subjective experience through an inter-subjective process has been recently examined by Holmes and Nolte (2019) from the perspective of the Bayesian brain. They note that the development of the generative model occurs through the “borrowing” of the maternal brain and suggest that “this borrowed brain model introduces a vital interpersonal dimension to the Bayesian process” (p. 4). The baby thus internalizes the experience of maternal care in order to reduce the entropy: “these embodied gestures present a model of the infant from the caregiver’s perspective helping the child to integrate primary sensory signals […] into regularities of emotional and interpersonal consequences” (p. 4). Within psychodynamic therapies, a similar process emerges as the subject develops its capacity for psychic integration through the intersubjective relationship with the therapist. This relationship fosters the resurgence of a “we mode” (Frith, 2012) in which “two heads are better than one” given that the other can “know our self better than we can know ourselves” (p. 5)^34^. Thus, “one of the roles of psychotherapy is to reactivate this process” (p. 5) through the deployment of free association as the expression and elaboration of the intrapsychic dynamic. Free association might thereby emerge as the joint connection of two Bayesian brains progressively leading, through their synchrony, to the dissolution of boundaries. In this manner, the “therapeutic duet for one helps bind potentially disruptive free energy in creative ways” (p. 6).

Early traumatic experiences like, for instance, what Winnicott (1963) calls “primitive agonies” are not integrated because they induced too high levels of entropy and thereby could not be “bound” by the psyche for they were not sufficient sources of pleasure^35^. This failure of integration might lead to mechanisms beyond the pleasure principle, such as the repetition compulsion. The analytic work creates a regression to primary processes within the safe environment of therapy, which permits to deconstruct the cleavage resulting from such early traumatic experiences. Through the practice of free association, the patient may affectively experience these previously unmetabolized agonies. The regression to primary levels would also emerge through daydreaming^36^ simultaneous with the free association. Free association would thus connect primary and secondary processes through the modalities of psychic integration permitting the renewal of symbolization processes.

It is perhaps in the crossing from primary to secondary processes that therapeutic gains are the most significant. Following the large body of research already developed concerning primary intersubjectivity and transmodal processes (Stern, 2000; Trevarthen and Aitken, 2001; Beebe et al., 2016), we could call this process “symbolizing transmodality.” This notion relates to the way in which the psychotherapy allows for the associative transfer between the various forms of symbolization. It results from an intersubjective associativity, as it emerges from the relationship developed between clinician and patient. It explores the primary and preverbal modes of communication involved in the mother-baby relationship, including sequences of motions of the body, rhythms of speech, tone, voice, sounds, facial expressions, etc. (Stern, 2000). The symbolizing transmodality transforms what the subject tries to explore through another sensory aspect. This passage permits the subject to “restore” the symbolization process by using a different sensorial modality. Its function is to metaphorize the inner experience as it moves from the most primary and unconscious forms to the more secondary and conscious processes.

From this point of view the analytic session forms a containing space for an increase in free energy allowing the subject to safely make prediction errors and confront surprise effects. Hence this astonishing paradox, as already noted by Reik (1936), of the necessity for patients, as well as clinicians, to preserve the ability to be surprised during therapy^37^. Through their echoing – and their own negative capability (Bion, 1965) – clinicians will favor the effects of surprise in patients. In Friston’s model, the effects of surprise are usually avoided by the psyche because they signify a gap between the internal and external worlds. Within psychoanalytic therapies, however, psychic mechanisms of “surprise” are required. For example, transference can be considered as a prediction error since the subject “confuses” the clinician with the parental imago. This confusion nonetheless gradually allows the subject to refine its own internal model by managing to differentiate the clinician from this projection^38^. Throughout the sessions, effects of surprise may thus emerge as experiences of pleasure^39^, for they can take the shape of sudden awareness or “eureka moments” leading to a improvement of the generative model. Such experiences reveal a form of free association and creativity^40^. They allow the patient to organize a set of internal representations through an externalized object supporting the projection of internal associativity. An encounter with an external object or an Other – whose properties favor processes of symbolization – produce an original subjective experience. The initial experience is thus transferred into the object and allows the subject to benefit from the symbolizing transmodality process^41^.

## Conclusion

**FIGURE 3:**
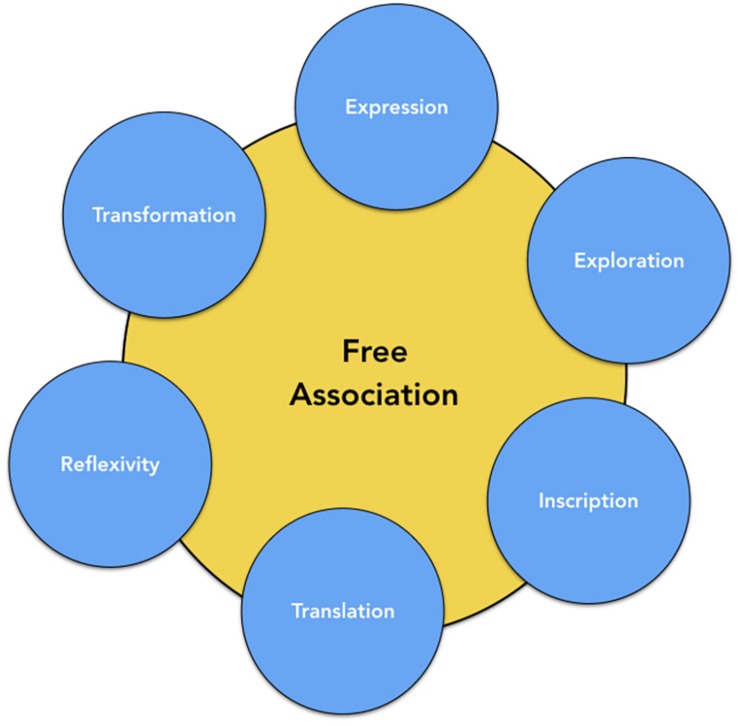
The different functions of free association.

During psychoanalytic and psychodynamic therapies, the patient passes from one idea to another and deploys a signifying chain composed of affects and representations using both verbal and non-verbal forms of expression. This free association process is an essential component of psychoanalytic practices and relies on complementary functions as illustrated in Figure 3. First, free association lets the subject express its intrapsychic world through increased focus on the internal experience and decreased focus on the environment. It allows for the exploration of intrapsychic reality – as a virtual reality generator (Hopkins, 2016) – by both the patient and the therapist, for the latter is also in a specific state of mental free association. Akin to dreams, free association also allows for the emergence of latent contents related to significant and mysterious elements of the subject’s psychic life. In this sense, it permits one to recognize the traces of traumatic experiences having induced “permanent disturbances of the manner in which the energy operates” (Holmes and Nolte, 2019, p. 6) as well as the traces left by the repression of certain drives^42^. Thus, as Bion (1962, 1965) suggests, the dream-like state that accompanies the free association helps the patient to transform non-thinkable thoughts into thinkable thoughts. This work of psychic integration through free association relies on a dynamic of “un-translation-translation” (Laplanche, 1987) in order to foster a more global and coherent elaboration of psychic experiences. Thus, free association becomes an essential tool for the synthesis of the ego. It refines the subject’s reflexivity as the subjectivation process develops and fosters symbolization of unelaborated traumatic experiences.

As proposed in this paper, this psychoanalytic understanding of free association also shares a number of theoretical parallels with contemporary neuroscientific models. As Scarfone (2018) suggests, “the contemporary usage of Helmholtzian ideas in brain science does indirectly support and justify recurring to free association in psychoanalysis” (p. 468). The activity of thought appears to correspond to a biological and psychological organization at primary and secondary levels whose cognitive correlates may be found in Kahneman (2011) System 1 and System 2. For Freud, a two-level division of mental processing (primary and secondary processes) is associated with the passage of “free energy” to “bound energy” according to a complex and hierarchical organization. In Solms’ model of the psyche, there is indeed first a primary form of consciousness which functions mainly through affects and an unbridled associativity. The second psyche’s main function is to form a coherent representation of the world using secondary and tertiary processes (Solms and Panksepp, 2012). Each of these levels works to limit the effects of surprise and disorganization. The transition between these levels of consciousness and their modes of associativity could emerge through the DMN whose purpose is to integrate and organize internal and external information. As Cieri and Esposito (2019) suggest: “Freudian constructs of the primary and secondary processes seem to have neurobiological substrates, consistent with self-organized activity in hierarchical cortical systems, and Freudian descriptions of the ego are consistent with the functions described of the DMN with its reciprocal exchanges with subordinate limbic and paralimbic brain systems” (p. 12). The role of psychoanalytic therapy, therefore, appears to be the reestablishment of this integrative work.

To conclude, we propose two further avenues of research that may be useful in orienting future work about free association. First, why is there a need for “associative transference” (from self to object or from self to other) for the subject to metabolize and integrate certain psychic contents? Current research about synesthesia – from the Greek “sunaisthesis” or simultaneous perception – may answer this question by addressing the unusual association of various senses. For instance, according to such research, a subject – notably individuals with autism (Neufeld et al., 2013) – may visually perceive colors associated with specific musical notes, numbers, or alphabetical letters. An immediate and automatic association from one sensory perception to another may thus emerge, which cerebral development would normally inhibit (Ward, 2013). Such form of “primordial associativity” may remain partly present in the psyche, while the symbolizing transmodality would emerge as its vestige. Innovative research joining, for instance, clinical and cognitive paradigms related to phenomena of synesthesia may thus lead to better understandings of free association and symbolization processes.

A second avenue might concern the limits of epistemological approaches to psychic states as they emerge thanks to free association. Psychoanalysts suggest that free association does not solely operate as a work of synthesis of the ego, but also as a work of disintegration of subjective experience. Thus, Scarfone (2018) reminds that “the lysis part of analysis literally means unbinding” (p. 473). The dissolution, or unbinding, work of analysis might be compared metaphorically to the nuclear fission defining the release of energy produced by the division of a heavy nucleus. Similarly, the work of analysis might release unbound energy through the free association process. For Barratt (2017), “herein lies the distinction between our discipline and all the therapies that prioritize the discourse of synthesis and integration” (p. 48). Furthermore, one may never represent the entirety of unconscious psychic activity, as free association is also a contact of the unknown. Thus, from an ontic perspective, “the praxis of free association effects an *ontic* change – a transmutation in the being of the subjective – which is not going to be explainable epistemologically.” (p. 49). For the same reason, Scarfone (2018) claims that “the repressed unconscious will never be fully transmuted into ego” (p. 476) and a part of the subjective experience will always remain as “non-representation” (David, 1992). Thus, there would be a risk in trying to explain or “rationalize” everything that is proposed by the patient^43^ and it could lead to pseudo-advances in the therapeutic process (Stern, 2011). Barratt (2017) also underlines that, from a clinical point of view, “the implication is that whereas one might become able to listen to the voicing of the repressed, through the praxis of free association, it is not to be assumed that the meaningfulness of the repressed is entirely translatable into the languages of representation” (p. 42). Something might have to remain obscure – what Freud calls the dream’s navel – and untranslatable into representable thoughts. Thus, Barratt (2017) continues, one part of the “repressed unconscious necessarily remains unknown (that is, unrepresentable), precisely because it involves impulses that are ontologically different from the meaningfulness of representationally” (p. 42). Such reflections might suggest the necessity to examine how far the connections between neuroscience and psychoanalysis might go and to what extent there may also be, for epistemological reasons, fundamental differences in regards to the knowledge emerging from these two complementary domains.

## Author Contributions

TR wrote the first draft of the manuscript. CM helped to translate and improve the quality of the writing of the manuscript.

## Conflict of Interest

The authors declare that the research was conducted in the absence of any commercial or financial relationships that could be construed as a potential conflict of interest.

## Footnotes

1 For an overall examination of Freud’s approach to the binding processes and their relationship toward the limitation of free association processes, see Holt (1962), who underlines how “Freud used binding (and its opposites, freedom or mobility of cathexis) in over a dozen different ways as his theory developed” (p. 522).

2 We will use in this paper the term “associativity” which is a translation of the French term “associativité” used in particular by Roussillon (2011). Associativity is a more general term than free association in the sense that it supposes that the free association process is not reducible to the verbal free association. The latter can take different forms according to various clinical devices: it can be, for instance, focal when it is centered on a dream or a projective test; it can involve several people when it arises from the “associative chain of a group;” and it can be “projected” externally on an object (for example, a painting) during artistic creation used during therapy (Rabeyron, 2017) leading to what Brun (2014) named “associativity of the shapes.”

3 For instance: a baseball bat and a ball cost $1.10. The bat costs $1.00 more than the ball. How much is the ball? Even among students from the Ivy Leagues, one out of two individuals responded erroneously (the correct answer is $0.05).

4 Some experiments led by Kahneman confirm the influence of subtle details on the activity of thought, in particular within System 1, and the modes of associativity. For instance, different images placed in front of a donation box will impact the amount of money perceived. Such experiments demonstrate that priming can have an impact on the associativity of thought that is automatic and unconscious.

5 Actually, entropy could be the fundamental principle that Freud (1920) was looking for in order to describe some inherent properties of living organisms. Entropy produces at the same time energy and excitation (pleasure drive) but can also induce destructivity and, finally, death (death drive) if not contained enough by the organism. This is an important topic from a conceptual point of view that would deserve further development.

6 Originating from Thomas Bayes, Bayesian statistics consist in deducing probabilities in response to past events. This model now appears more and more frequently within the field of empirical psychology (see Dienes, 2011).

7 From this point of view, The Free Energy Principle also appears as a way to formalize the notion of autopoiesis and the relationship between the organism and the environment (Friston, 2013).

8 The relationship with the external world can also be shaped by the actions of the biological organism, actions that can lead to variations in the potential effects of entropy (Friston, 2009).

9 The ego thus emerges as “an associative structure occupying the higher level of organization of the generative model, that comes to influence lower levels of the hierarchy” (Connolly and van Deventer, 2017, p. 14).

10 This model appears similar to the work of Aulagnier (1975) distinguishing between originary, primary, and secondary levels to describe the constant work of psychic metabolization underlying representational activity.

11 On the Markov blanket, Cieri and Esposito (2019) note that “self-organizing systems must have a specific identifiable boundary condition: the so-called Markov blanket, which acts as a protective screen, […] as a veil through which we are able to recognize and distinguish an internal side from an external environment of an organism, inferring the external or internal causes of sensations, perceptions, or changes” (p. 4). As Mellor (2018) suggests, Markov blankets seem to fall under what psychoanalysts have named psychic envelopes, a notion which appears notably in the work of Anzieu (1974) following Bion (1965) writings on the distinction between the container and the contained and the idea of a “membrane” separating conscious and unconscious processes.

12 Panksepp (2010) describes three levels of control in the brain’s emotion-affective processing: primary process (basic-primordial affect), secondary process emotions (learning processes associated with the basal ganglia) and tertiary process (corresponding to neocortical awareness functions). These three different levels are associated with three different levels of consciousness (Solms and Panksepp, 2012): anoetic, noetic, and autonoetic. Consciousness and its relation to emotions are thus considered as the consequence of hierarchic models in which anoetic would correspond to primary processes while noetic and autonoetic would rather correspond to secondary processes in Freud’s model (Solms and Panksepp, 2012, p. 151). The origins of consciousness thus appear at an anatomical subcortical level which corresponds to seven primary processes shared by all mammals: seeking, lust, care, play, rage, fear, and panic. Therefore, Solms and Panksepp argue that “all consciousness ultimately derives from upper brainstem sources” (p. 163) and that consciousness is, metapsychologically, “generated in the id” (p. 164). Consequently, consciousness initially emerges distinctly from reflexive processes and the anoetic level probably emerged before the noetic and autonoetic levels.

13 Similar claims have also been brought forward by Damasio (2010) who proposes a distinction between the proto-self, the core consciousness, and the extended consciousness. See Rabeyron (2016) for more details concerning parallels between Damasio’s model and psychoanalytical models of consciousness.

14 That is, the neurological structure of the brainstem, influenced by somatic and emotional stimuli responsible for muscle tone and vigilance states.

15 Solms relies on data originating from neuroscientific research on hydranencephaly, a condition marked by the destruction of the cerebral cortex *in utero* (2013, p. 10), where one can nonetheless notice all signs of a primary form of consciousness.

16 Solms and Panksepp (2012) propose that secondary processes derive anatomically from the stabilization of representations by the cortex. The tertiary processes Panksepp describe correspond to Freud’s secondary processes for they use “word-representations” whose “symbolic nature enables to represent abstract relations between the concrete objects of thoughts” (p. 166).

17 It should be mentioned here that the variation in global cerebral activity from focus on a task to the resting state is only 5% (Raichle and Snyder, 2007), which may suggest that psychic work can largely operate independently of the environment.

18 The attention system is not unified. It is divided between *salient system* and *dorsal attention system*. Their hierarchic relation is established at the higher level of the DMN, while they themselves occupy intermediary levels.

19 The transition from an affective to a stabilized form of consciousness is described by Solms as the transition from primary to secondary processes (Solms, 2013) or from primary–secondary processes to tertiary processes (Solms and Panksepp, 2012) depending on which model he relies on (mainly Freudian or Panksepp models). As in Solms (2013), we will use the distinction between primary and secondary processes to describe this transition in the following pages.

20 Regarding linguistic developments stemming from bodily schemas, see notably Lakoff and Johnson (2003). Language emerges from metaphoric and metonymic processes – as Lacan (1966) earlier suggested – derived from physical experiences. Each level of the generative model is thus influenced by the logics and peculiarities of the lower levels.

21 Hobson et al. (2014) and Hopkins (2016) also argue that primary processes form an “innate virtual reality” that characterizes dream processes and a high degree of entropy.

22 The ability to contain uncertainty is an essential component of Bion (1965) theories. The latter argues that symbolization processes require a “negative capability” (a term borrowed from the poet Keats). Within psychoanalysis and psychodynamic therapies, this expression signifies that, to integrate an experience, the subject must first be able to deconstruct it in order to rebuild it differently. This process of deconstruction and construction requires the subject to tolerate uncertainty, represented by the negative capability (Rabeyron, 2018). In other words, the deployment of the association necessitates the capacity to accept uncertainty in order to catalyze symbolization.

23 Depression appears more precisely as a mechanism of withdrawal and disinvestment from the environment that allows the subject to reduce the states of excitement stemming from interactions with the outside world. The depressed subject is frequently confronted with psychic conflicts increasing free energy. The withdrawal of the depressed subject thus offers a solution to reduce the entropy. Yet, this solution has the disadvantage of preventing the implementation of adaptive strategies. A withdrawal process of a different nature also emerges in psychosis (De Masi et al., 2015).

24 In psychosis, we witness the disintegration of the psychic “membrane” separating conscious and unconscious processes when the “disobjectalizing function” of the death drive described by Green pervades the psyche (Mellor, 2018). The formation of the symbol, an essential component of psychic functioning, is no longer operational and the words are treated as things within the “symbolic equation” described by Anna Segal (1957).

25 While it is important, from a theoretical perspective, to distinguish these two modes of functioning and their neurobiological correlates, one must also bear in mind that they probably work in parallel. Kahneman (2011) research indeed demonstrates that System 1 and System 2 often work in concert.

26 As their name indicates, extreme forms of symbolization designate the extreme and unusual expressions of the subject’s psychic integration and transformation. They emerge, notably, during altered states of consciousness (Cardeña and Winkelman, 2011) and various anomalous or exceptional experiences (Rabeyron et al., 2010; Rabeyron and Loose, 2015). In relation to Friston’s theories, extreme forms of symbolization may correlate with a state of global reorganization of the generative model.

27 The notion of symbolization deserves some precisions (see also, Roussillon, 2015). The word “symbol” originates from the Greek *sumbolon* and the verb *sumballesthai*, which signifies “bringing together.” In ancient Greek, the symbol defines a broken shard of pottery whose parts are given to a contractor, the latter ensuring the authenticity of the “contract” by reassembling the pieces. More specifically, the term symbolization designates the operation through which one thing represents another. In a clinical sense, the symbolization defines the various steps that permit the subject to transform “primary psychic matter” into a state of reflectivity and subjectivity. In other words, the symbolization designates the many ways the subject transforms its experience in order to integrate and appropriate it subjectively. From the perspective of psychopathology, a variety of sufferings may be understood as “blockages” of symbolization processes. This is why it is important to understand the modalities and logics of symbolization. We distinguish more precisely between primary or archaic symbolization, which Freud designates as thing-representation, and secondary symbolization, which Freud calls word-representation.

28 Within therapy, these symbolization processes were developed through clinical encounters with borderline and narcissist personalities (Green, 1990). Such encounters require therapists and patients to work at very primary levels of psychic functioning which are hardly reached through verbal or linguistic expression. This is why the expression of associativity has been developed by Roussillon (2015) to underscore the importance of mediations for the patient’s elaboration of traumas dividing the personality.

29 The verbal expression of free association emerges from other forms of associativity. Thus, the spontaneous free play of the baby (Pikler, 1962), which passes from one object to another in the exploration of the surrounding world, appears as a preform of free associativity. We first learn to play – to associate – with objects before we begin to play with words. Yet, the verbal mode of free association does not entail the disappearance of this first mode of free association. This explains why a non-verbal form of free association emerges in the clinical setting and why the therapeutic relationship must be attuned to this other form of associativity.

30 This process finds echo in the work of Friston and Frith (2015) examining how the generative model can be improved, thanks to synchronization processes, as seen at a behavioral level by the simulation of birdsongs. These synchronization processes are particularly complex because “we are trying to infer how our sensations are caused by others, while they are trying to infer our behaviors” (p. 1). In this regard, “communication facilitates long-term changes in generative models that are trying to predict each other” (p. 12). If these two generative models are close enough in their functioning and share a collective narrative (in psychotherapy, the same language and the same culture), they will progressively become attuned and each of them will develop a generative model with better predictability (and consequently a reduction of free energy). During this synchronization process, hidden states will emerge that belong to *both* birds which could correspond to what is described notably by Ogden (1994) or Green (2002) as the analytical third and tertiary processes. In other words, this research suggests that synchronization processes are essential in order to improve generative models, a discovery that may underline a fundamental aspect of the psychotherapeutic process.

31 César and Sara Botella have approached the question of psychic figurability through regression and hallucinatory states as a “work of transformation directed toward the implementation of a psychical intelligibility and heterogeneity” (Botella, 2006, p. 5). See also Botella and Botella (2001).

32 It is not, however, a question of reducing the alpha function to a neurobiological network, but of supposing that properties of psychic functioning arise from this level of biological organization, while being irreducible to biological processes. On this epistemological topic, see the analyses of Connolly and van Deventer (2017).

33 Green (1995) notably calls “tertiary processes” the back and forth movements, or the binding processes, between primary and secondary processes, thus complicating Freud’s binary model by emphasizing its interconnections (Green, 2005). In this perspective, both the alpha function and the DMN appear to fall under tertiary processes.

34 In the same way that we cannot see our own face directly – while the other can – our own psychic reality seems, in certain respects, more accessible to others. For instance, others are generally better placed to tell us if we have some unusual element on our face. It is probably similar with psychotherapy, which might explain why, from an evolutionary perspective, the intersubjective relation is more efficient for the auto-representation of processes.

35 This hypothesis comes from Freud (1920) who supposes that an experience needs enough pleasure to be integrated in psychological reality. A traumatic experience (leading to PTSD for example), if it is too painful, will not be integrated. From the FEP perspective, we can consider that the traumatic experience induces too much entropy – or excitation – to be integrated by the generative model of the brain. It will then be cleaved from the functioning of the generative model.

36 Much neuroscientific research has examined Random Episodic Silent Thinking (REST) (Andreasen et al., 1995) and Mind-Wandering (Mooneyham and Schooler, 2013). While we may not address these here, they also present interesting parallels with states of daydreaming as understood in psychoanalysis.

37 In psychoanalysis, the dynamic of transference is therefore understood to generate effects of surprise that can later lead to positive outcomes. This is, notably, an important aspect distinguishing psychoanalysts from cognitive and behavioral therapists, particularly within the medical field, where the clinician will work – at the opposite – to avoid or diminish the effects of surprise.

38 To suggest a simple metaphor, just as, in skiing, one may only progress by learning to fall, in psychoanalysis, one must be allowed to make “mistakes” otherwise unacceptable in daily life.

39 Perhaps the surprise might also induce pleasure when an increase in entropy leads to a reorganization of the generative model. On the other hand, when the degree of surprise is too great, we might assume that it cannot induce a reorganization and the experience therefore becomes unpleasant. Thus, as Holmes and Nolte (2019) suggest, “all depends on decoupling – introducing a degree of play into the Bottom Up/Top Down surprise –minimizing articulations of everyday life” (p. 8). The tact shown by the clinician might be related to this ability to induce an appropriate amount of surprise and to avoid the use of defense mechanisms as means of curbing an excessive entropy.

40 For example, in the field of science, Newton’s discovery of the laws of gravity under an apple tree, or Einstein’s theorization of special relativity after repeated observations of clocks and trains at Bern station, offer examples of this creative associativity that comes from transmodal experiences.

41 Clinical work with children, where the symbolic transmodality expresses itself essentially through play, offers an illustration of this process. Indeed, the work of “stabilization” of mental objects may be sub-operative, leading the child to “stick” the self to objects, as Winnicott (1958) suggested through the notions of “transitional space” and the “found-created.” Hence the complexity of the clinic with children, where the very first session will present the latter’s preoccupations, but in a condensed or compressed way. The child’s first play, in particular, condenses the associations relating to the causes of the child’s suffering, which often become intelligible only through multiple sessions.

42 From this point of view, what is most essential is not what the patient says, but what the patient does not say because the negation emerges as a consequence of defense mechanisms and repression.

43 Bion (1965) calls −K the link which does not permit the authentic integration of an experience. From a Bionian perspective, free association is a means to establish a contact with O a an unattainable Real that underlies biological and psychic dimensions.

## References

[B1] Alberini C. M. Ansermet F. Magistretti P. (2013). “Memory reconsolidation, trace reassociation and the Freudian unconscious,” in *Memory Reconsolidation*, ed. Alberini C. M. (New York, NY: Academic Press), 293–310.

[B2] Andreasen N. C. O’Leary D. S. Cizadlo T. Arndt S. Rezai K. Watkins G. L. (1995). Remembering the past: Two facets of episodic memory explored with positron emission tomography. *Am. J. Psychiatr.* 152 1576–1585. 7485619 10.1176/ajp.152.11.1576

[B3] Anzieu D. (1974). Le moi-peau. *Nouv Rev. Psychanal.* 9 195–208.

[B4] Aulagnier P. (1975). *La violence de L’interprétation (2003 Edn).* Paris: Puf.

[B5] Barratt B. B. (2016). *Radical Psychoanalysis: An Essay on Free-Associative Praxis.* London: Routledge.

[B6] Barratt B. B. (2017). Opening to the otherwise: the discipline of listening and the necessity of free−association for psychoanalytic praxis. *Int. J. Psychoanal.* 98 39–53. 10.1111/1745-8315.12563 27469203

[B7] Beebe B. Messinger D. Margolis A. Buck K. A. Chen H. (2016). A systems view of mother-infant face-to-face communication. *Dev. Psychol.* 52 556–571. 10.1037/a0040085 26882118 PMC4808406

[B8] Bion W. (1965). *Transformations: Passage de l’apprentissage à la croissance* (2002 Edn). Paris: PUF.

[B9] Bion W. R. (1962). *Aux Sources de L’expérience* (2003 Edn). Paris: PUF.

[B10] Bollas C. (2008). *The Evocative Object World.* London: Routledge.

[B11] Botella C. (2006). *Rêverie-Rêverie et Travail de Figurabilité. Table Ronde, Débats Sans Frontières.* Paris: Société Psychanalytique de Paris.

[B12] Botella C. Botella S. (1990). “La problématique de la régression formelle de la pensée et de l’hallucinatoire,” in *La psychanalyse: Questions Pour Demain*, ed. Schimmel I. (Paris: PUF).

[B13] Botella C. Botella S. (2001). *La Figurabilité Psychique (2007 Edn).* Paris: in press.

[B14] Brun A. (2014). Médiation thérapeutique picturale et associativité formelle dans les dispositifs pour enfants avec troubles envahissants du développement. *La Psychiatr. Enfant* 57 437–464.

[B15] Buckner R. L. Andrews-Hanna J. R. Schacter D. L. (2008). The brain’s default network: anatomy, function, and relevance to disease. *Ann. N. Y. Acad. Sci.* 1124 1–38. 10.1196/annals.1440.011 18400922

[B16] Cardeña E. Winkelman M. (2011). *Altering Consciousness: Multidisciplinary Perspectives.* London: Praeger.

[B17] Carhart-Harris R. L. Friston K. J. (2010). The default-mode, ego-functions and free-energy: a neurobiological account of freudian ideas. *Brain* 133 1265–1283. 10.1093/brain/awq010 20194141 PMC2850580

[B18] Carhart-Harris R. L. Leech R. Hellyer P. J. Shanahan M. Feilding A. Tagliazucchi E. (2014). The entropic brain: a theory of conscious states informed by neuroimaging research with psychedelic drugs. *Front. Hum. Neurosci.* 8:20. 10.3389/fnhum.2014.00020 24550805 PMC3909994

[B19] Cieri F. Esposito R. (2019). Psychoanalysis and neuroscience: the bridge between mind and brain. *Front. Psychol.* 10:1790. 10.3389/fpsyg.2019.01983 31555159 PMC6724748

[B20] Connolly P. van Deventer V. (2017). Hierarchical recursive organization and the free energy principle: from biological self-organization to the psychoanalytic mind. *Front. Psychol.* 8:1695. 10.3389/fpsyg.2017.01695 29038652 PMC5623195

[B21] Damasio A. R. (2010). *L’autre Moi-Même les Nouvelles Cartes du Cerveau, de la Conscience et Des Émotions.* Paris: O. Jacob.

[B22] David C. (1992). *La Bisexualité Psychique.* Paris: Payot.

[B23] De Masi F. Davalli C. Giustino G. Pergami A. (2015). Hallucinations in the psychotic state: psychoanalysis and the neurosciences compared. *Int. J. Psychoanal.* 96 293–318. 10.1111/1745-8315.12239 25327380

[B24] de M’Uzan M. (1994). *La Bouche de L’inconscient.* Paris: Gallimard.

[B25] Dienes Z. (2011). Bayesian versus orthodox statistics: which side are you on? *Perspect. Psychol. Sci.* 6 274–290. 10.1177/1745691611406920 26168518

[B26] Dimkov P. R. (2019). Large-scale brain networks and freudian ego. *Psychol. Thought* 12 14–27. 10.5964/psyct.v12i2.328 20194141

[B27] Donnet J. L. (2012). Le procédé et la règle: l’association libre analytique. *Rev. Fr. Psychanal.* 76 695–723.

[B28] Eschel O. (2006). Where are you, my beloved? On absence, loss, and the enigma of telepathic dreams. *Int. J. Psychoanal.* 87 1603–1627. 10.1516/7gm3-mldr-1w8k-lvlj 17130085

[B29] Evrard R. Rabeyron T. (2012). Les psychanalystes et le transfert de pensée: enjeux historiques et actuels. *Evol. Psychiatr.* 77 589–598. 10.1016/j.evopsy.2012.05.002

[B30] Freud S. (1895). *Project for a Scientific Psychology. Standard Edition of Complete Works*, Vol.I. London: Hogarth Press.

[B31] Freud S. (1900). *The Interpretation of Dreams. Standard Edition of Complete Works*, Vol.IV-V. London: Hogarth Press.

[B32] Freud S. (1901). *The psychopathology of everyday life. Standard Edition of Complete Works*, Vol.IV-V. London: Hogarth Press.

[B33] Freud S. (1905). *Fragment of an Analysis of a Case of Hysteria. Standard Edition of Complete Works*, Vol.VII. London: Hogarth Press.

[B34] Freud S. (1909). *The Rat Man. Standard Edition of Complete Works*, Vol.X. London: Hogarth Press.

[B35] Freud S. (1913). *On the beginning of treatment. Standard Edition of Complete Works*, Vol.XII. London: Hogarth Press.

[B36] Freud S. (1915). *The Unconscious. Standard Edition of Complete Works*, Vol.XIV. London: Hogarth Press.

[B37] Freud S. (1920). *Beyond the Pleasure Principle. Standard Edition of Complete Works*, Vol.XVIII. London: Hogarth Press.

[B38] Freud S. (1930). *Civilization and its Discontents. Standard Edition of Complete Works*, Vol.XXI. London: Hogarth Press.

[B39] Freud S. (1937). *Constructions in Analysis. Standard Edition of Complete Works*, Vol.XXIII. London: Hogarth Press.

[B40] Freud S. (1941). *Findings, Problems, Ideas. Standard Edition of Complete Works*, Vol.XXIII. London: Hogarth Press.

[B41] Freud S. Breuer J. (1895). *Studies in Hysteria. Standard Edition of Complete Works*, Vol.II. London: Hogarth Press.

[B42] Friston K. (2009). The free-energy principle: a rough guide to the brain? *Trends Cogn. Sci.* 13 293–301. 10.1016/j.tics.2009.04.005 19559644

[B43] Friston K. (2013). Life as we know it. *J. R. Soc. Interface* 10 20130475. 10.1098/rsif.2013.0475 23825119 PMC3730701

[B44] Friston K. Frith C. (2015). A duet for one. *Conscious. Cogn.* 36 390–405. 10.1016/j.concog.2014.12.003 25563935 PMC4553904

[B45] Frith C. D. (2012). “Implicit metacognition and the we-mode,” in *Paper perended at Workshop on “Pre-reflective and Reflective Processing in Social Interaction* (Cambridge: Clare College, University of Cambridge).

[B46] Georgieff N. (2010). Psychanalyse, neurosciences et subjectivités. *Neuropsychiatr. Enfance Adolesc.* 58 343–350.

[B47] Green A. (1990). *La Folie Privée.* Paris: Gallimard.

[B48] Green A. (1995). Note sur les processus tertiaires. *Rev. Fr. Psychanal.* 36 151–155. 4650061

[B49] Green A. (2002). *Idées Directrices Pour une Psychanalyse Contemporaine.* Paris: PUF.

[B50] Green A. (2005). *Key Ideas for a Contemporary Psychoanalysis.* New York, NY: Routledge.

[B51] Hobson J. Allan, Hong Charles C.-H. Friston Karl J. (2014). Virtual reality and consciousness inference in dreaming. *Front. Psychol.* 5:1133. 10.3389/fpsyg.2014.01133 25346710 PMC4191565

[B52] Holmes J. Nolte T. (2019). Surprise” and the bayesian brain: implications for psychotherapy theory and practice. *Front. Psychol.* 10:592. 10.3389/fpsyg.2019.00592 30984063 PMC6447687

[B53] Holt R. R. (1962). A critical examination of Freud’s concept of bound vs. *Free cathexis*. *J. Am. Psychoanal. Assoc.* 10 475–525. 10.1177/000306516201000302 14036323

[B54] Hopkins J. (2016). Free energy and virtual reality in neuroscience and psychoanalysis: A complexity theory of dreaming and mental disorder. *Front. Psychol.* 7:922. 10.3389/fpsyg.2016.00922 27471478 PMC4946392

[B55] Kahneman D. (2003). A perspective on judgment and choice: mapping bounded rationality. *Am. Psychol.* 58 697–720. 10.1037/0003-066x.58.9.697 14584987

[B56] Kahneman D. (2011). *Thinking, Fast and Slow.* New York, NY: Farrar, Straud and Giroux.

[B57] Lacan J. (1966). *Écrits.* Paris: Le seuil.

[B58] Lakoff G. Johnson M. (2003). *Metaphors we Live by.* Chicago: University of Chicago Press.

[B59] Laplanche J. (1987). *Nouveaux Fondements pour la Psychanalyse.* Paris: Puf.

[B60] Leuzinger-Bohleber M. Kaufhold J. Kallenbach L. Negele A. Ernst M. Keller W. (2019). How to measure sustained psychic transformations in long-term treatments of chronically depressed patients: symptomatic and structural changes in the LAC Depression Study of the outcome of cognitive-behavioural and psychoanalytic long-term treatments. *Int. J. Psychoanal.* 100 99–127. 10.1080/00207578.2018.1533377 33945717

[B61] Lothane H. Z. (2018). Free association as the foundation of the psychoanalytic method and psychoanalysis as a historical science. *Psychoanal. Inquiry* 38 416–434. 10.1080/07351690.2018.1480225

[B62] Magistretti P. Ansermet F. (2010). *Neurosciences et Psychanalyse.* Paris: Odile Jacob.

[B63] Mancia M. (2006). Implicit memory and early unrepressed unconscious: their role in the therapeutic process (How the neurosciences can contribute to psychoanalysis). *Int. J. Psychoanal.* 87 83–103. 10.1516/d43p-8upn-x576-a8v0 16635862

[B64] Méheust B. (1999). *Somnambulisme et Mediumnité.* Paris: Les Empêcheurs de penser en rond.

[B65] Mellor M. J. (2018). Making worlds in a waking dream: where bion intersects friston on the shaping and breaking of psychic reality. *Front. Psychol.* 9:1674. 10.3389/fpsyg.2018.01674 30319479 PMC6171158

[B66] Mooneyham B. W. Schooler J. W. (2013). The costs and benefits of mind-wandering: a review. *Can. J. Exp. Psychol.* 67 11. 10.1037/a0031569 23458547

[B67] Neufeld J. Roy M. Zapf A. Sinke C. Emrich H. M. Prox-Vagedes V. (2013). Is synesthesia more common in patients with Asperger syndrome? *Front. Hum. Neurosci.* 7:847. 10.3389/fnhum.2013.00847 24367321 PMC3856394

[B68] Ogden T. H. (1994). The analytical third: working with intersubjective clinical facts. *Int. J. Psycho. Anal.* 75 3–20. 8005761

[B69] Panksepp J. (1998). *Affective neuroscience: The Foundations of Human and Animal Emotions.* Oxford: Oxford UP.

[B70] Panksepp J. (2010). Affective neuroscience of the emotional BrainMind: evolutionary perspectives and implications for understanding depression. *Dialogues Clin. Neurosci.* 12 533–545. 21319497 10.31887/DCNS.2010.12.4/jpanksepp PMC3181986

[B71] Panksepp J. Solms M. (2011). What is neuropsychoanalysis? Clinically relevant studies of the minded brain. *Trends Cogn. Sci.* 16 6–8. 10.1016/j.tics.2011.11.005 22153583

[B72] Parat C. (2013). L’affect p1artagé. *Rev. Fr. Psychosom.* 44 167–182. 19668536

[B73] Pikler E. (1962). *Que sait Faire Votre Bébé?.* Paris: Les éditeurs français réunis.

[B74] Rabeyron T. (2016). Les processus de symbolisation et de représentation comme espace transitionnel pour la psychanalyse et les neurosciences. *Evol. Psychiatr.* 81 160–175. 10.1016/j.evopsy.2015.03.003

[B75] Rabeyron T. (2017). Médiations thérapeutiques et processus de symbolisation : de l’expérience sensible à la modélisation. *Evol. Psychiatr.* 82 351–364. 10.1016/j.evopsy.2017.01.001

[B76] Rabeyron T. (2018). Constructions finies et constructions infinies: de l’épistémologie psychanalytique dans ses rapports à la vérité. *Analysis* 2 143–155. 10.1016/j.inan.2018.07.002

[B77] Rabeyron T. (2019). Processus transformationnels et champ analytique: un nouveau paradigme pour les modèles et les pratiques cliniques. *Evol. Psychiatr.* (in press).

[B78] Rabeyron T. Chouvier B. Le Maléfan P. (2010). Clinique des expériences exceptionnelles: du trauma à la solution paranormale. *Evol. Psychiatr.* 75 633–653. 10.1016/j.evopsy.2010.09.004

[B79] Rabeyron T. Loose T. (2015). Anomalous experiences, trauma, and symbolization processes at the frontiers between psychoanalysis and cognitive neurosciences. *Front. Psychol.* 6:1926. 10.3389/fpsyg.2015.01926 26732646 PMC4685320

[B80] Raichle M. E. Snyder A. Z. (2007). A default mode of brain function: a brief history of an evolving idea. *Neuroimage* 37 1083–1090. 10.1016/j.neuroimage.2007.02.041 17719799

[B81] Ramstead M. J. Kirchhoff M. D. Friston K. J. (2019). A tale of two densities: Active inference is enactive inference. *Adap. Behav.* 1–15. PMC7418871 32831534

[B82] Reik T. (1936). *Surprise and the Psycho-Analyst: On the Conjecture and Comprehension of Unconscious Processes, 2014 Edn.* New York: Routledge.

[B83] Roussillon R. (1992). *Du Baquet de Mesmer au Baquet de Sigmund Freud.* Paris: PUF.

[B84] Roussillon R. (2001). *Le Plaisir et la Répétition: Théorie du Processus Psychique.* Paris: Dunod.

[B85] Roussillon R. (2009). L’associativité. *Libr. Cah. Psychanal.* 20 19–35.

[B86] Roussillon R. (2011). *La Disposition D’esprit Clinique. In: Manuel de Pratique Clinique.* Paris: Elsevier.

[B87] Roussillon R. (2012). L’associativité polymorphique et les extensions de la psychanalyse. *Carnet. Psy.* 162 27–31.

[B88] Roussillon R. (2015). An introduction to the work on primary symbolization. *Int. J. Psychoanal.* 96 583–594. 10.1111/1745-8315.12347 26173881

[B89] Roussillon R. Chabert C. Ciccone A. Ferrant A. (2007). *Manuel de Psychologie et Psychopathologie Clinique Générale.* Paris: Masson.

[B90] Scarfone D. (2018). Free association, surprise, trauma, and transference. *Psychoanal. Inquiry* 38 468–477. 10.1080/07351690.2018.1480232

[B91] Segal H. (1957). Note on symbol formation. *Int. J. Psychoanal.* 38 395–401. 13501926

[B92] Shedler J. (2010). The efficacy of psychodynamic psychotherapy. *Am. Psychol.* 65 98–109. 10.1037/a0018378 20141265

[B93] Solms M. (2013). The conscious id. *Neuropsychoa* 15 5–19. 10.1080/15294145.2013.10773711

[B94] Solms M. (2018). The hard problem of consciousness and the free energy principle. *Front. Psychol.* 9:2714. 10.3389/fpsyg.2018.02714 30761057 PMC6363942

[B95] Solms M. Panksepp J. (2012). The “Id” knows more than the “Ego” admits: Neuropsychoanalytic and primal consciousness perspectives on the interface between affective and cognitive neuroscience. *Brain Sci.* 2 147–175. 10.3390/brainsci2020147 24962770 PMC4061793

[B96] Solms M. Turnbull O. (2011). What is neuropsychoanalysis? *Neuropsychoa* 13 133–145. 10.1080/15294145.2011.10773670

[B97] Steinert C. Munder T. Rabung S. Hoyer J. Leichsenring F. (2017). Psychodynamic therapy: as efficacious as other empirically supported treatments? A meta-analysis testing equivalence of outcomes. *Am. J. Psychiatr.* 174 943–953. 10.1176/appi.ajp.2017.17010057 28541091

[B98] Stern A. (2011). Investigation psychanalytique sur le groupe borderline des névroses. Quelle thérapie engager?. *Rev. Fr. Psychanal.* 75 331–348.

[B99] Stern D. N. (2000). *The Interpersonal World of the Infant: A View From Psychoanalysis and Developmental Psychology.* New York, NY: Basic Books.

[B100] Stern D. N. (2004). *The Present Moment in Psychotherapy and Everyday Life.* New York, NY: Norton.

[B101] Trevarthen C. Aitken K. (2001). Infant intersubjectivity: research, theory, and clinical application. *J. Child Psychol. Psychiatr.* 42 3–48. 10.1111/1469-7610.00701 11205623

[B102] Ward J. (2013). Synesthesia. *Annu. Rev. Psychol.* 64 49–75. 10.1146/annurev-psych-113011-143840 22747246

[B103] Widlöcher D. (1996). *Les Nouvelles Cartes de la Psychanalyse.* Paris: Odile Jacob.

[B104] Widlöcher D. (2010). Distinguishing psychoanalysis from psychotherapy. *Int. J. Psychoanal.* 91 45–50. 10.1111/j.1745-8315.2009.00233.x 20433471

[B105] Winnicott D. W. (1958). *Through Paediatrics to Psycho-analysis.* London: Karnac.

[B106] Winnicott D. W. (1963). *Fear of breakdown. Psychoanalytic Explorations (1974 Edn).* Boston: Harvard UP.

[B107] Woll C. F. J. Schönbrodt F. D. (2019). A series of meta-analytic tests of the efficacy of long-term psychoanalytic psychotherapy. *Eur. Psychol.* 25 51–72.

[B108] Yovell Y. Solms M. Fotopoulou A. (2015). The case for neuropsychoanalysis: Why a dialogue with neuroscience is necessary but not sufficient for psychoanalysis. *Int. J. Psychoanal.* 96 1745–1553. 10.1111/1745-8315.12332 26227821

